# CLRM-12. HYBRID DESIGNS FOR USING EXTERNAL CONTROLS IN PHASE 3 GLIOBLASTOMA TRIALS

**DOI:** 10.1093/noajnl/vdab112.011

**Published:** 2021-09-21

**Authors:** Mei-Yin Polley, Daniel Schwartz, James Dignam

**Affiliations:** University of Chicago, Chicago, IL, USA

## Abstract

While recent Phase 3 glioblastoma (GBM) trials have failed to establish novel therapies, they potentially provide a high-quality source of external control patients treated with temozolomide. We consider hybrid two-stage adaptive designs that leverage these external controls to safely accelerate Phase 3 GBM trials. The basic strategy is that first patients are randomized 1:1 between the control and experimental arms, then an interim check measures similarity between the trial's control patients and potential external controls, and finally if this interim similarity is high the randomization ratio is changed accordingly and the external controls are used in the final analysis. An extensive simulation study is conducted to assess operating characteristics and we discuss when these hybrid designs can accelerate GBM therapy development while maintaining strict error control.

